# Precision and safety of Multilevel Cervical Transpedicular Screw Fixation with 3D Patient-Specific Guides; A Cadaveric Study

**DOI:** 10.1038/s41598-019-51936-w

**Published:** 2019-10-30

**Authors:** Andrea Sallent, Manuel Ramírez, Jordi Catalá, Alfonso Rodríguez-Baeza, Joan Bagó, Matías de Albert, Roberto Vélez

**Affiliations:** 1 Orthopaedic Department, Hospital Vall d’Hebron. Universitat Autónoma de Barcelona, Barcelona, Spain; 2 Institut de Recerca Vall d’Hebron (VHIR), Barcelona, Spain; 3 Radiology Department, Institut Guirado, Barcelona, Spain; 4 Department of Morphological Science, Universitat Autonoma de Barcelona, Barcelona, Spain; 5 Radiology Department, Vall d’Hebron University Hospital, Barcelona, Spain

**Keywords:** Bone, Three-dimensional imaging, Preclinical research

## Abstract

The aim is to design a patient-specific instrument (PSI) for multilevel cervical pedicle screw placement from C2 to C7, as well as verifying reliability and reproducibility. Computed tomography (CT) scans were obtained from 7 cadaveric cervical spines. Using Mimics software, semiautomatic segmentation was performed for each cervical spine, designing a 3D cervical spine bone model in order to plan transpedicular screw fixation. A PSI was designed according to the previously cited with two cannulated chimneys to guide the drill. The guides were 3D printed and surgeries performed at the laboratory. Postoperative scans were obtained to study screw placement. Sixty-eight transpedicular screws were available for study. 61.8% of all screws were within the pedicle or partially breached <4 mm. No differences were observed between cervical levels. None of these screws had neurovascular injury. Of the 27 screws with a grade 3 (screw outside the pedicle; 39.7%), only 2 had perforation of the transverse foramen and none of them would have caused a neural injury. In conclusion, multilevel PSI for cervical pedicle screw is a promising technology that despite showing improvements regarding free-hand technique requires further studies to improve the positioning of the PSI and their accuracy.

## Introduction

Transpedicular screw fixation was first described in 1989 for the upper cervical spine^1,2^, and five years later for the middle and lower cervical spine for traumatic lesions^3^. Regarding posterior stabilization of the cervical spine, pedicle screw fixation has shown to be a better biomechanical choice than lateral mass screws^4^. Pedicle screws show an improved biomechanical stability over lateral mass screws, allowing for shorter instrumentations with improved reposition capacities^5,6^. However, safety concerns regarding injury to the vertebral artery, spinal cord or nerve root have been reported^7,8^. Furthermore, cervical pedicle diameters are smaller than those in the thoracic or lumbar spine, increasing its technical difficulty.

In a cadaveric study using the funnel technique and without computed assistance, authors reported 17% of pedicle perforations^9^. Ludwig *et al*. observed that 65.5% of transpedicular screws had a critical breach when placed only with morphometric data, 39.6% if a laminoforaminotomy was performed to place the screws and decreased to 10.6% if using computed-assisted navigation^10^.

Currently, free-hand is the most widely used technique for cervical pedicle screw placement, with an inaccurate placement range of 10–40% according to the literature^11^. Several techniques have been described in order to improve the pedicle screw placement^5,10,12–14^. Richter *et al*. described a navigation system (Vector Vision; BrainLAB AG, Heimstetten, Germany) that improved screw placement compared to a conventional technique^5^. However, the same authors pointed out that being a computed aid, the system could crash due to several errors, or be imprecise due to the reference clamp. In studies were CT navigation was used, the percentage of screws exceeding 4 mm of violation was only 3.3%, whereas studies using fluoroscopy alone, 40% had screw violation exceeding 4 mm, in thoracic spine^14^. The small dimensions of the spinous processes, especially in the middle cervical spine, makes the position of the reference clamps difficult^5^. Ludwig *et al*. found an improvement with computer-assisted surgery as above mentioned^10^. Reinhold *et al*. described a custom-made aiding frame in combination with conventional fluoroscopy that again improved the cervical pedicle screw placement^12^. A more recent study defined a 3D locator guide that was superior to manual manipulation of cervical screws^13^. Despite navigation systems have shown high reliability, anyone using such a system should be aware of its inconvenient. First, the system could crash because of the hardware, software or human failure. Secondly, the surgeon should not rely entirely in the virtual information and make his/her own verification. Last but not least, the high cost of navigated systems make it difficult for every spine surgeon to have access everywhere. Thus, other alternatives have been considered to substitute navigation systems.

A patient-specific instrument (PSI) is a personalized tool that guides the saw, chisel, or drill in a specific, pre-designed cutting path. It has been previously been described for orthopaedic procedures as well in spine surgery^11,15,16^. This tool offers the advantage of being less expensive and less complex than navigated or robotic surgery. Several studies have described the use of patient-specific screw guides for cervical spine in one to two levels^11,17–19^. However, our aim is to describe the hypothetical advantages of multilevel screw guides for cervical spine. Surgical time would be hypothetically faster if we use one multilevel cervical guide instead of seven single-level guides. Furthermore, printing cost and time could be significantly reduced if only one guide is printed. Theoretically, if only one multilevel guide could be used for the entire cervical spine; both time and cost could be reduced, as printing one PSI instead of 7 would be cheaper, and avoiding changing guide every level would reduce time too.

Until now, limited studies have reported the use of 3D PSIs in cervical spine, however, to our knowledge, none using a multi-level drill guide template that can assure satisfactory accuracy of cervical pedicle screw placement.

The objective of the current study is to design a patient-specific instrument for multilevel cervical pedicle screw placement from C2 to C7, as well as verifying reliability and reproducibility.

Our hypothesis is that the new techniques of software and 3D modeling can allow the design and manufacturing of a patient-specific instrument safely and reproducibly for multilevel cervical pedicle screws positioning.

## Results

Sixty-eight pedicles were available for placing the screws and further studying the results. Table 1 shows the distribution of screw positioning according to the grade classification used in the present study by Rajasekaran *et al*.^20^. Mann-Whitney U test was used to study differences in number of screws between right or left side, as well as within grade classification, showing non-significant differences (p 0.764).

**Table 1:** Distribution of screws according to the classification used in the present study.

			Side		Total
			Right	Left	
Classification	Grade 0 (normal)	Total count	12	12	24
		% classification	50%	50%	100%
		% side	33.3%	37.5%	35.3%
		% total	17.6%	17.6%	35.3%
	Grade 1 (<2 mm)	Total count	4	2	6
		% classification	66.7%	33.3%	100%
		% side	11.1%	6.2%	8.8%
		% total	5.9%	2.9%	8.8%
	Grade 2 (2–4 mm)	Total count	5	6	11
		% classification	45.5%	54.5%	100%
		% side	13.9%	18.8%	16.2%
		% total	7.4%	8.8%	16.2%
	Grade 3 (outside)	Total count	15	12	27
		% classification	55.6%	44.4%	100%
		% side	41.7%	37.5%	39.7%
		% total	22.1%	17.6%	39.7%
Total		Total count	36	32	68
		% classification	52.9%	47.1%	100%
		% side	100%	100%	100%
		% total	52.9%	47.1%	100%

First line (total screws) shows the absolute number of screws according to every side and grade classification. % classification refers to the % of right, left or bilateral screws within that grade classification. % side mentions the % of right, left or bilateral screws according to the total number. % total defines the % of screws within the total number of screws. P value 0.764.

Secondly, the distribution of screws according to the cervical level was studied (Table 2). Despite C6 and C7 had more screws classified as grade 3, no statistical differences were observed between the different cervical levels and the screw classification grade (Kruskal –Wallis test, p 0.535). Interestingly, 2 of the specimens had all screws placed within the pedicle, and most of grade 2 and 3 were in the same specimen. However, no statistical differences were found between cervical specimens.

**Table 2:** Distribution of screws according to the cervical level p value 0.535.

			Classification				Total
			0 (normal)	1 (<2 mm)	2 (2–4 mm)	3 (outside)	
Cervical level	C2	Total count	5	2	1	2	10
		% level	50%	20%	10%	20%	100%
		% classification	20.8%	33.3%	9.1%	7.4%	14.7%
		% total	7.4%	2.9%	1.5%	2.9%	14.7%
	C3	Total count	3	2	3	2	10
		% level	30%	20%	30%	20%	100%
		% classification	12.5%	33.3%	27.3%	7.4%	14.7%
		% total	4.4%	2.9%	4.4%	2.9%	14.7%
	C4	Total count	5	2	1	5	13
		% level	38.5%	15.4%	7.7%	38.5%	100%
		% classification	20.8%	33.3%	9.1%	18.5%	19.1%
		% total	7.4%	2.9%	1.5%	7.4%	19.1%
	C5	Total count	4	0	2	6	12
		% level	33.3%	0.0%	16.7%	50%	100%
		% classification	16.7%	0.0%	18.2%	22.2%	17.6%
		% total	5.9%	0.0%	2.9%	8.8%	17.6%
	C6	Total count	5	0	1	7	13
		% level	38.5%	0.0%	7.7%	53.8%	100%
		% classification	20.8%	0.0%	9.1%	25.9%	19.1%
		% total	7.4%	0.0%	1.5%	10.3%	19.1%
	C7	Total count	2	0	3	5	0.10
		% level	20%	0.0%	30%	50%	100%
		% classification	8.3%	0.0%	27.3%	18.5%	14.7%
		% total	2.9%	0.0%	4.4%	7.4%	14.7%
Total		Total count	24	6	11	27	68
		% level	35.3%	8.8%	16.2%	39.7%	100%
		% classification	100%	100%	100%	100%	100%
		% total	35.3%	8.8%	16.2%	39.7%	100%

61.8% of all screws were within the pedicle or partially breached <4 mm. No differences were observed between cervical levels. None of these screws had neurovascular injury. Of the 27 screws with a grade 3 (39.7%), only 2 had perforation of the transverse foramen and none of them would have caused a neural injury.

## Discussion

Our study shows that although multilevel patient-specific instruments have better outcomes than free-hand technique in pedicle screw placement of cervical spine, further improvements need to be addressed in order to achieve better outcomes.

The accuracy of free-hand placement has been previously studied, with numbers around 55–65%^13,21,22^. Single-level PSIs have the highest accuracy numbers, around 95–100% according to the previously published^11,17^. Accuracy of the multilevel guides is 65%, of the misplaced screws none caused neurovascular injury. Manual placement varies significantly depending on the surgeon’s experience and preferred insertion method. Intraoperative imaging has improved screw accuracy^23^. However, these systems have several limitations, such as radiation (both for the patient and surgical team), occupy a larger space, and restrict the working space for surgeons^23^.

Two of 68 screws placed in the present study were in close contact to the vertebral artery. Vertebral arterial injury during cervical spine surgery is relatively low but may be associated with serious complications such as arteriovenous fistulae, late-onset bleeding, pseudoaneurysm and thrombosis with embolic incidents, cerebral ischemia, stroke and even death^24^. The reported rate of neurovascular injury in cervical spine surgery is approximately 5%^8,25^. In the present study, 2.9% of all screws were in close contact to the vertebral artery, and despite a CT angiography is mandatory to rule out any possible lesion, apparently no injury to the vertebral artery was caused. However, as stated before, perforation of the transverse foramen (as measured in the present study) does not necessarily cause vertebral artery injury^21^. A CT angiography should have been performed to rule out the possible vascular injury.

Several limitations have to be reviewed. First, ours is an *in vitro* study with no control group. Furthermore, the grading system available in the previous studies is arbitrary and varies from one study to another, making the comparison hard^11,12,20,26^. Despite the figures showed in the present study are not as promising as the previously stated studies, ours is the first study to use a multilevel guide. Using a multilevel guide has not been previously described probably due to the high difficulty of designing a guide that is perfectly adaptable to more than two cervical levels. Fluoroscopy was not used during the entire surgical process of the present study, which could have advised of screw mal-positioning. However, the aim of the multilevel PSI guide is to be sufficiently reliable in order to avoid fluoroscopy during surgery.

Another possible reason for our higher perforation rates may be the cadavers used. All specimens had degenerative changes in the vertebrae, which even made the identification of landmarks difficult. Additionally, the specimens’ bones were osteoporotic, which made perforation of the cortex of the transverse foramen more plausible. Undoubtedly, the screw diameter could have influenced the perforation rate, considering that some pedicles were wider than the screw diameter. In the present study, screw diameter was of 3.5 mm, the standard screw diameter.

A limitation of the multilevel PSI is being able of reproducing *in vivo* the 3D reconstruction that has been manufactured. Dissection errors are presumably the same with a multilevel guide than with a single level, even with 7 single-level it would seem easier to be mistaken. If a thorough dissection of the posterior structures of the vertebra has not been achieved, the guide fails to adapt intimately to the posterior bone surface, leading to screw deviation. Furthermore, printing 1 PSI instead of 7 could be cheaper and presumably faster.

There are several advantages of PSI technology: custom-made for every patient, cheap, relative short learning curve, few requirements of material, less radiation, and does not limit the surgeon’s work space^27^. In fact, in the present study, one of the surgeons was a non-spine surgeon with no previous experience in cervical spine surgery in order to minimize bias from experience.

3D printing in orthopedics is still not commonplace. Its potential as a tool to help plan surgery is immense. In further studies with multilevel guides, the fitting of the template to the cervical spine should be improved. In our study, the spinous process was used as reference point.

In conclusion, multilevel patient-specific instruments for cervical pedicle screw is a promising technology that despite showing improvements regarding free-hand, requires further studies to improve the positioning of the PSI and therefore improve their accuracy. A further study with limited drill bits and measured lengths should be performed.

## Methods

**Figure 1:**
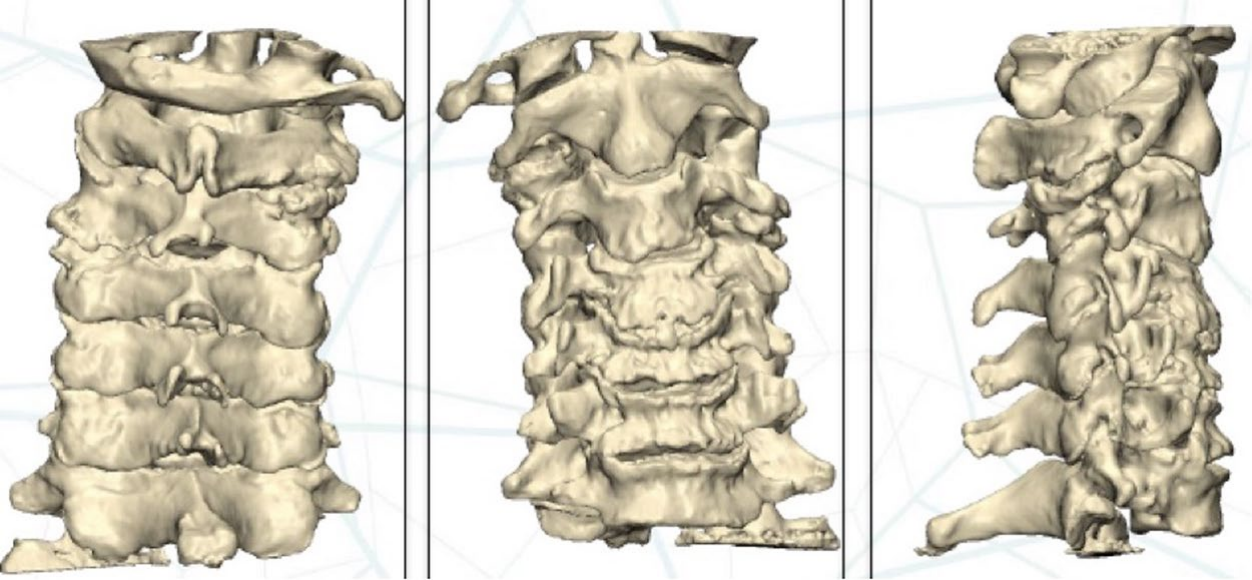
Preoperative 3D planning. From left to right, anterior view, posterior view and lateral view.

**Figure 2:**
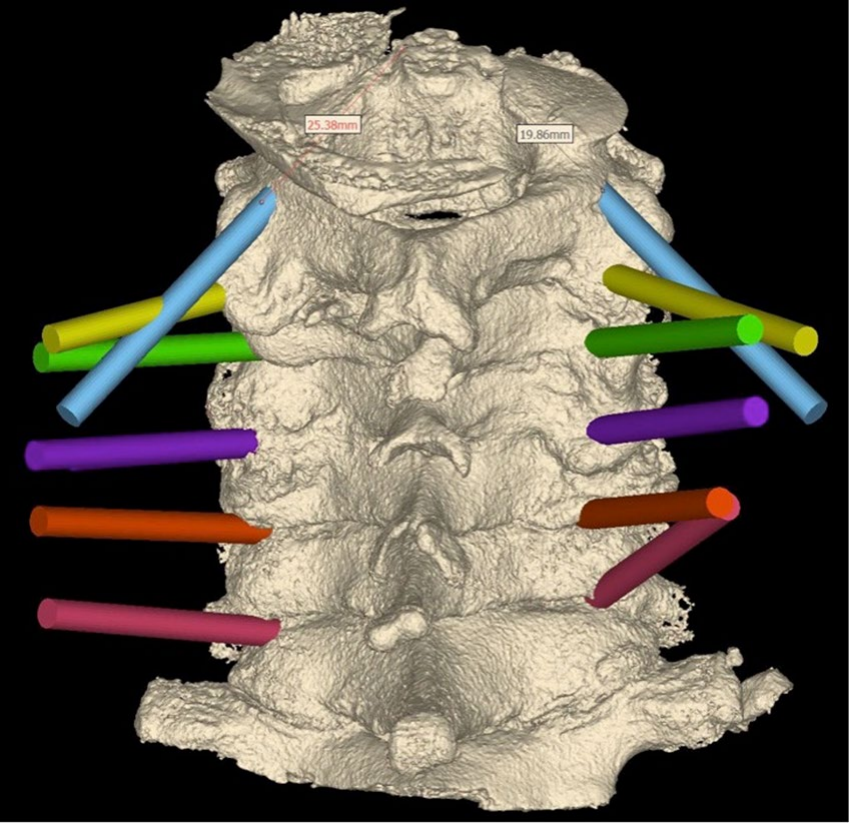
Planning of the screw tunnels, each colour represents a cervical level.

**Figure 3:**
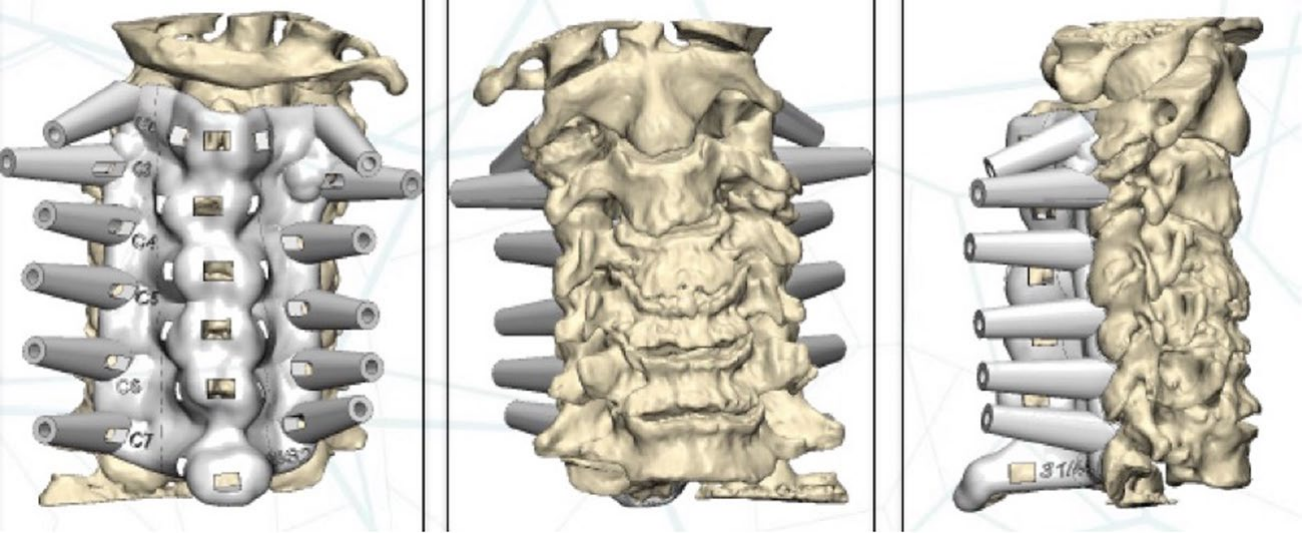
Computer 3D planning of the templates’ design. Anterior, posterior and lateral view (from left to right).

An experimental study was performed using 7 cadaveric cervical spines from the Anatomy Laboratory. The study was carried out in accordance with the guidelines and local regulations. The Ethics Committee on Animal and Human Experimentation approved the experimental protocol (procedure 2904). The cadaveric pieces had no prior surgeries. The cervical spines were scanned (Aquilion Vision, Toshiba, Irvine, CA, USA) in 0.5-mm slices. DICOM files were then exported to the Mimics software (Materialise, Belgium). Semiautomatic segmentation was then performed for each spine, and a 3D cervical spine bone model was created (1:1) (Fig. 1). Next, using 3-matic software (Materialise, Belgium), pedicle screw fixation was planned (Fig. 2). The PSI was then designed to place the screws in the desired position and to anatomically adapt to the bone surface (Fig. 3). Two cannulated chimneys were designed to guide the drill through the previously designed path. The guides were 3D-printed (Avinent Implant System, S.L., Santpedor, Spain) using an EOS-Formiga P110 printer in polyamide (PA2200). Each PSI was then packaged with an identification number that correlated with the specific cervical spine for which it had been designed.

**Figure 4:**
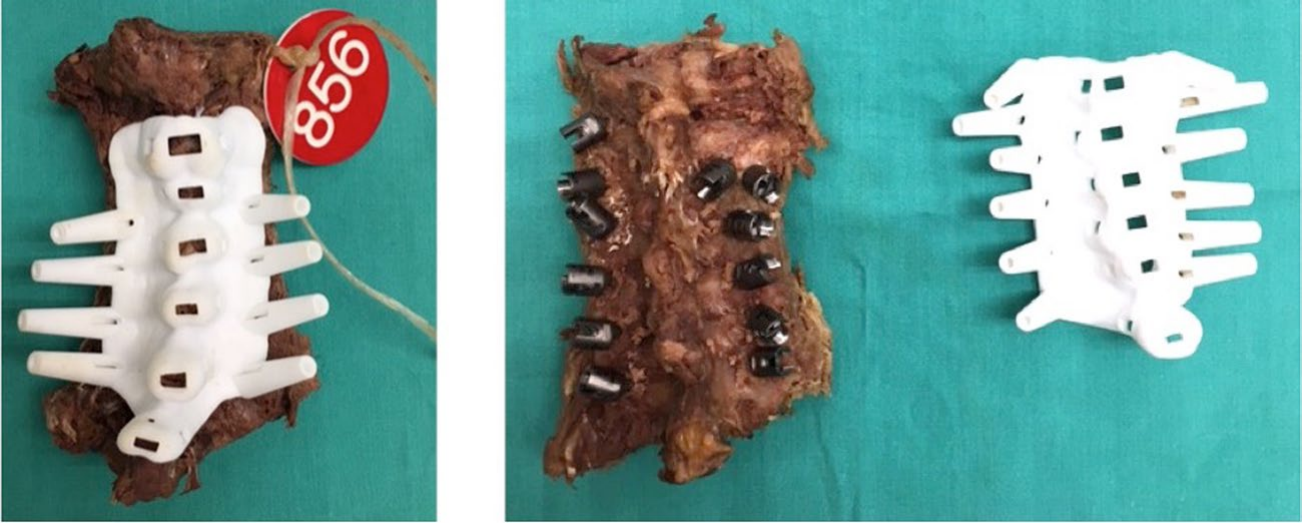
Intraoperative images of the templates. Left image; example of how the template adapt to the cadaveric cervical column.

Surgeries were carried out in the Anatomy Laboratory. Two surgeons performed all surgeries; an experienced cervical spine surgeon and an orthopedic resident, in order evaluate the easiness of the procedure. A standard posterior approach was performed, exposing adequately the spinous process and lateral margin of the lateral mass. The Orthofix Ascent ^TM^ LE POCT system was used in all cervical spines (Fig. 4). The template was considered correctly placed if there was no movement with digital pressure. The fitting of the template was designed looking for support over the spinous process, which had to be well exposed during the approach as well as the lateral edge of the articular process. The template was used to drill the screw tunnel and then was removed to place the screw manually. Bone specimens were marked, identified and packaged in transparent transport plastic bags. Aquilion Vision (Toshiba, Irvine, CA, USA) was used to scan the specimens, with 0.5-mm slices. DICOM files were then exported to the Mimics software (Materialise, Belgium). Next, semi-automatic segmentation was performed on each specimen and 3D models were created (1:1).

Two independent radiologists measured the screw position following the grading system described by Rajasekaran *et al*.^20^:

- Grade 0: screw is centered in the pedicle with only plastic deformation of the pedicle cortex. No pedicle perforation
- Grade 1: screw threads or less than 2 mm of the screw cross-section penetrates the cortex. No contact of the screw with neurovascular structures.
- Grade 2: the core screw diameter is outside the pedicle 2–4 mm but there is no contact with neurovascular structures.
- Grade 3: the screw is completely outside the pedicle.

Non-parametric tests were used to compare differences between sides and cervical levels, as the sample did not follow a normal distribution (Mann-Whitney *U* and Kruskal-Wallis tests for independent samples).
