# Author Correction: Precision and safety of Multilevel Cervical Transpedicular Screw Fixation with 3D Patient-Specific Guides; A Cadaveric Study

**DOI:** 10.1038/s41598-020-59139-4

**Published:** 2020-02-06

**Authors:** Andrea Sallent, Manuel Ramírez, Jordi Catalá, Alfonso Rodríguez-Baeza, Joan Bagó, Matías de Albert, Roberto Vélez

**Affiliations:** 1 Orthopaedic Department, Hospital Vall d’Hebron. Universitat Autónoma de Barcelona, Barcelona, Spain; 2 Institut de Recerca Vall d’Hebron (VHIR), Barcelona, Spain; 3 Radiology Department, Institut Guirado, Barcelona, Spain; 4 Department of Morphological Science, Universitat Autonoma de Barcelona, Barcelona, Spain; 5 Radiology Department, Vall d’Hebron University Hospital, Barcelona, Spain

Correction to: *Scientific Reports* 10.1038/s41598-019-51936-w, published online 30 October 2019

The Acknowledgements section in this Article is incomplete.

“Authors would like to special thank Instituto Guirado (Barcelona, Spain), for performing all CT scans for the present study. Authors would like to thank Avinent Implant System, S.L., Santpedor, Spain for printing the 3D guides.”

should read:

“Authors would like to special thank Instituto Guirado (Barcelona, Spain), for performing all CT scans for the present study. Authors would like to thank Avinent Implant System, S.L., Santpedor, Spain for printing the 3D guides. Authors would like to appreciate MBA Institut for their help with the surgical material and during the surgical procedure.”

